# Renal vascular resistance is increased in patients with kidney transplant

**DOI:** 10.1186/s12882-019-1617-2

**Published:** 2019-11-27

**Authors:** Johanna Päivärinta, Vesa Oikonen, Anne Räisänen-Sokolowski, Tuula Tolvanen, Eliisa Löyttyniemi, Hidehiro Iida, Pirjo Nuutila, Kaj Metsärinne, Niina Koivuviita

**Affiliations:** 1 Department of Nephrology, Turku University Hospital, PL 52,Kiinanmyllykatu 4-8, 20521 Turku, Finland; 2 Department of Medicine, University of Turku, Turku, Finland; 3 Turku PET Centre, University of Turku, Turku, Finland; 4 Department of Pathology, Helsinki University Hospital and Helsinki University, Helsinki, Finland; 5 Department of Medical Physics, Turku University Hospital, Turku, Finland; 6 Department of Biostatistics, University of Turku, Turku, Finland

**Keywords:** Kidney transplant, Renal perfusion, Kidney biopsy, Positron emission tomography, Chronic kidney disease

## Abstract

**Background:**

Despite improvement in short-term outcome of kidney transplants, the long-term survival of kidney transplants has not changed over past decades. Kidney biopsy is the gold standard of transplant pathology but it’s invasive. Quantification of transplant blood flow could provide a novel non-invasive method to evaluate transplant pathology. The aim of this retrospective cross-sectional pilot study was to evaluate positron emission tomography (PET) as a method to measure kidney transplant perfusion and find out if there is correlation between transplant perfusion and histopathology.

**Methods:**

Renal cortical perfusion of 19 kidney transplantation patients [average time from transplantation 33 (17–54) months; eGFR 55 (47–69) ml/min] and 10 healthy controls were studied by [^15^ O]H_2_O PET. Perfusion and Doppler resistance index (RI) of transplants were compared with histology of one-year protocol transplant biopsy.

**Results:**

Renal cortical perfusion of healthy control subjects and transplant patients were 2.7 (2.4–4.0) ml min^− 1^ g^− 1^ and 2.2 (2.0–3.0) ml min^− 1^ g^− 1^, respectively (*p* = 0.1). Renal vascular resistance (RVR) of the patients was 47.0 (36.7–51.4) mmHg mL^− 1^min^− 1^g^− 1^ and that of the healthy 32.4 (24.6–39.6) mmHg mL^− 1^min^−1^g^−1^ (*p* = 0.01). There was a statistically significant correlation between Doppler RI and perfusion of transplants (*r* = − 0.51, *p* = 0.026). Transplant Doppler RI of the group of mild fibrotic changes [0.73 (0.70–0.76)] and the group of no fibrotic changes [0.66 (0.61–0.72)] differed statistically significantly (*p* = 0.03). No statistically significant correlation was found between cortical perfusion and fibrosis of transplants (*p* = 0.56).

**Conclusions:**

[^15^ O]H_2_O PET showed its capability as a method in measuring perfusion of kidney transplants. RVR of transplant patients with stage 2–3 chronic kidney disease was higher than that of the healthy, although kidney perfusion values didn’t differ between the groups. Doppler based RI correlated with perfusion and fibrosis of transplants.

## Introduction

Renal allograft survival has improved over past decades mainly because of increased first-year survival [1, 2]. However, long-term transplant outcome and yearly graft attrition rate of 2,5–5% has remained the same [1, 3, 4].

Changes in renal tissue oxygenation and microvasculature are considered as major determinants of progression of chronic kidney disease (CKD), irrespective of its cause [5, 6]. Microvascular and endothelial dysfunction has shown to be associated to kidney transplant failure, too [7]. In human allograft biopsy studies, especially angioregression and loss of peritubular capillaries seem to be associated with development of interstitial fibrosis and graft dysfunction [8–11].

Kidney biopsy, which is the gold standard of evaluation of transplant parenchyma, is invasive and prone to sampling errors [12]. On the other hand, creatinine is a late marker of kidney dysfunction. Assessment of transplant perfusion could perhaps be used to study kidney function.

Quantification of renal blood flow (RBF) has been challenging because of complicated vascular structure of the kidneys as well as difficulties in finding quantitative and noninvasive measuring techniques. Several semi-quantitative methods have been used to evaluate renal allograft perfusion like scintigraphy [13], dynamic tissue Doppler [14] and real-time contrast-enhanced sonography [15]. In recent years also magnetic resonance imaging (MRI) has provided new techniques to study transplant function [16–18].

Doppler-echo with resistance index (RI) is based on blood velocity and blood pressure, thus it measures perfusion indirectly. RI has been used to monitor allograft function at predetermined time points after transplantation as well as in acute settings. However, conflicting data has been reported of its value in reflecting allograft condition and histology [19–22].

[^15^O]H_2_O positron emission tomography (PET) is a non-invasive and quantitative method to measure regional one-kidney perfusion without contrast agent [23]. However, it has only been used in evaluation of perfusion of native kidneys, not in transplants [24–30]. Furthermore, the correlation between transplant perfusion and histology is not well established in humans [14, 31, 32]. In this pilot study our aim was to measure cortical perfusion of kidney transplants by means of [^15^O]H_2_O PET and compare transplant perfusion to transplant biopsy and Doppler RI.

## Methods

### Study subjects

Nineteen kidney transplant patients and 10 healthy controls were included in the study. Patients were recruited from the nephrology outpatient clinic of Turku University Central Hospital during 2017–2018. Kidney transplantations of the patients were performed between 1/2011–2/2017. There were 58 kidney transplantations during that period. Because our aim was to study microvascular function, only patients with no signs of cardiovascular, cerebrovascular or peripheral artery disease were selected from those 58 patients with kidney transplant. We also excluded patients with eGFR< 30 ml/min. Furthermore, patients with reduction of eGFR more than 20 ml/min between the time points of kidney biopsy and PET-imaging were excluded. None of the healthy controls had any sign of vascular disease or used any medication.

### Study design

PET-imaging and laboratory examinations were performed in all study subjects. Ambulatory 24 h blood pressure monitoring [33] was additionally assessed within 1 month after PET imaging during normal medication in transplant patients. Measuring of RI by Doppler echo [34] was a part of clinical routine follow up of transplant patients. The time interval between Doppler echo and PET imaging was on average 1–6 months. Protocol transplant biopsy was performed 1 year after the transplantation.

### PET-imaging

The imaging studies were carried out after a 10-h overnight fast. Caffeine and alcohol were prohibited for 1 day before assessment. Patients were instructed to take their medication as usually on study day except angiotensin converting enzyme (ACE)-inhibitors and angiotensin receptor blockers (ARB), which were discontinued 3 days before imaging.

The subjects were positioned supine in the camera [Discovery 690 PET/computed tomography (CT)] scanner, GE Medical Systems, Waukesha, Wisconsin, USA). Venous catheter was placed in an antecubital vein for injection of [^15^O]H_2_O. A low dose helical CT scan with automatic dose modulation (120 kVp, 10–80 mAs, noise index 30, pitch of 1.375, rotation time of 0.5 s) was acquired during normal breathing before the PET scan to correct for photon scatter and attenuation. Thereafter 700 MBq of [^15^O]H_2_O was given and PET scanning was conducted. Blood pressure and heart rate (HR) were measured by an automatic oscillometric blood pressure machine.

### Image processing and correction

The PET scan protocol consisted of 26 frames over a total of 360 s (15 × 4, 4 × 10, 4 × 20, 3 × 60). PET data were corrected for dead time, decay and measured photon attenuation. Scatter correction was limited. PET images were reconstructed using OSEM (VUE Point FX), with 2 iterations and 24 subsets, FOV 35 cm and matrix 256 X 256, and filter cutoff 2.0 mm.

### Calculation of renal blood flow

**Fig. 1:**
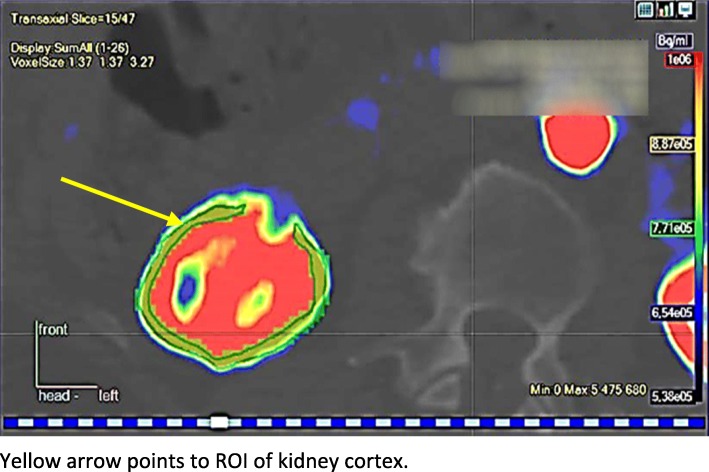
Cortex ROI in a transaxial slice of PET image

Regions of interest (ROI) for the whole cortical region of the kidney were drawn on a summed reconstructed image on an average of six coronal planes using Carimas software [35] (Fig. 1). For the calculation of renal perfusion from the PET study in the healthy, the input function was estimated using an average time activity curve (TAC) from descending aorta cavity ROIs [26] drawn on average 6 planes. ROI was drawn automatically around the aorta on static images using a threshold of 80% of the maximum activity for the aorta [26]. In transplant patients TAC was taken from external iliac artery because aorta was not seen undivided in the scanning field of kidney transplant (Additional files 1, 2, 3 and 4).

Renal perfusion was estimated from renal cortex and image-derived input function by nonlinear fitting of a one-tissue compartmental model [30] with four parameters: K_1_, k_2_, V_a_, and delay, where K_1_ and k_2_ are the unidirectional transport rates of [^15^O]H_2_O into and from tissue, V_a_ is the vascular volume fraction, and delay parameter accounts for the difference of radioactivity appearance times between the blood and tissue curves. K_2_ multiplied by physiological partition coefficient (p_phys_) is renal perfusion (ml min^− 1^ g^− 1^) [30]. Renal vascular resistance (RVR) was calculated according to formula: mean arterial pressure (MAP)/renal perfusion.

### Renal arterial resistance index and 24 h ambulatory blood pressure monitoring

RI was assessed by means of Doppler ultrasound [34] in patients with kidney transplant 1–6 months before PET-imaging. Doppler RI was calculated according to formula: (peak systolic velocity-end diastolic velocity)/peak systolic velocity.

Twenty-four hour ambulatory blood pressure [33] was monitored during normal medication 0–4 weeks after PET-imaging. MAP was calculated according to formula: diastolic blood pressure + (systolic blood pressure-diastolic blood pressure)/3.

### Laboratory tests

P-creatinine and U-Na were taken on the day of PET imaging. P-creatinine was used in assessment of renal function based on eGFR equation from The Chronic Kidney Disease Epidemiology Collaboration (CKD-EPI)-study [36]. Sodium balance, which is known to affect renal perfusion was estimated by measurement of U-Na.

### Kidney transplant biopsy

Transplant protocol biopsy was taken 1 year after kidney transplantation. An experienced nephropathologist evaluated the biopsies according to Banff classification [37, 38]. We assessed modified Banff’s fibrosis score [39] which was calculated after the sum of gs + ci + ct from the biopsy report. Gs corresponded to the degree of glomerulosclerosis (0, no global gs; 1, up to 25% gs; 2, 26–50% gs; and 3, > 50% gs) and ci and ct corresponded to interstitial fibrosis and tubular atrophy as classified in Banff [37, 38]. Thereby maximum score was 9. Due to minor sclerotic changes in kidney biopsies (score 0–4/9) we combined scores 0 and 1 for a group of no fibrosis and scores 2–4 for a group of mild fibrosis.

For evaluation of inflammatory changes we assessed an inflammation score which was calculated after the sum of g + i + t + ti from the biopsy report according to Banff classification [37, 38]. G corresponded to glomerulitis, i to inflammation in unscarred cortical parenchyma, t to tubulitis and ti to total inflamed cortical parenchyma including scarred sections. Hence maximum score was 12. Because of minor inflammatory changes (score 0–6/12) in biopsies we combined scores 0 and 1 for a group of no inflammatory changes and scores 2–6 for a group of mild inflammatory changes.

### Statistics

Comparison between healthy control subjects and kidney transplant patients was performed for categorical variables with Fisher’s exact test (gender) and one-way analysis of variance (for variables following normal distribution) or Wilcoxon rank sum test (for non-normal data) for continuous variables. Same analysis method was used to compare variables with no inflammatory changes and mild inflammatory changes. In addition, Pearson correlation coefficients were calculated when association were examined. All statistical tests were performed as 2-tailed, with a significance level set at 0.05. The analyses were performed using SAS System, version 9.4 for Windows (SAS Institute Inc., Cary, NC, USA).

## Results

### Study subjects

Causes of CKD were as follows: 6 IgA nephropathies, 4 type I diabetic nephropathies, 1 lupus nephritis, 4 autosomal dominant polycystic kidney diseases, 2 medullary cystic kidney diseases, 1 FSGS and 1 kidney disease without a specific diagnosis. One of the patients had a kidney-pancreas transplantation.

All the kidney transplant patients were on antihypertensive medication. Seven of 19 patients had either ACE-inhibitor or ARB. Calcium channel blocker was used by 16 patients, beta blocker by 15 and diuretic by 8. There were 3 patients who had a combination of 4 antihypertensives and 4 patients who had a combination of 3 antihypertensives. Statins were used by 11 patients.

Four patients used a combination of tacrolimus, mycophenolate and corticosteroid as immunosuppressive medication, seven patients used a combination of cyclosporine, mycophenolate and corticosteroid, a combination of cyclosporine and mycophenolate was used by four patients and a combination of tacrolimus and mycophenolate by four patients. Fourteen patients were in peritoneal dialysis (PD) and 4 in hemodialysis (HD) before kidney transplantation. One patient had gradus IA and one patient had gradus IIA rejection during early period after transplantation.

The control subjects didn’t use any medication. The demographics of the study subjects are shown in the Table 1.

**Table 1:** Baseline characteristics

	Kidney transplant patients (*N* = 19)	Controls (*N* = 10)
Age (years)	52 (23–70)	56 (48–64)
BMI (kg/m2)	28 (24–32)	25 (23–27)
Sex F/M (N)	10/9	7/3
eGFR on the day of PET-imaging (ml/min)	55 (47–69)*	82 (79–87)
eGFR on the day of kidney biopsy (ml/min)	60 (57–72)	
P-Crea on the day of PET-imaging (umol/l)	115 (97–131)*	73 (72–86)
U-Na (mmol/l)	63 (42–116)	58 (47–81)
fP-chol (mmol/l)	4.8 (4.1–5.5)	
fP-LDL (mmol/l)	2.7 (2.4–3.3)	
fP-HDL (mmol/l)	1.5 (1.3–1.8)	
fP-Tg (mmol/l)	1.3 (1.0–1.8)	
B-Hb (g/l)	141 (131–148)	
fP-gluk (mmol/l)	5.5 (5.1–6.2)	
U-prot (g/l)	0	
Hypertension (N)	19	0
DM I/II (N)	4/0	0
Smoking (N)	0	0
Time in dialysis (months)	15 (12–27)	0
Age of kidney transplant (months)	33 (17–54)	

Values are median (Q1-Q3) except age, which is expressed as median (Q0-Q4)

*BMI* Body mass index

*p < 0.05 controls versus kidney transplant patients

Age, BMI and gender were similar in the healthy controls and kidney transplant recipients (*p* > 0.05 in all). There was a statistically significant difference in eGFR and creatinine between controls and transplant patients (*p* < 0.0001 both).

### Hemodynamics

Hemodynamic parameters are shown in Table 2.

**Table 2:** Hemodynamic parameters

	Kidney transplant patients *N* = 19	Controls *N* = 10
Blood pressure (mmHg) on study day		
Systolic	153 (137–160)*	126 (123–133)
Diastolic	80 (76–85)	75 (70–79)
MAP	105 (99–107)*	93 (88–96)
Heart rate (beats/min)	63 (50–76)	56 (50–60)
24 h ambulatory blood pressure (mmHg)		
Systolic	144 (136–148)	
Diastolic	81 (75–86)	
MAP	102 (96–108)	
Heart rate (beats/min)	68 (56–72)	

Values are median (Q1-Q3)

*MAP* Mean arterial pressure

*P < 0.05 controls versus kidney transplant patients

Transplant patients had statistically significantly higher systolic blood pressure and MAP than the healthy controls (*p* = 0.0004; *p* = 0.0019).

### Renal perfusion

Renal perfusion values are shown in Table 3. There was no statistically significant difference in renal perfusion values between the healthy and kidney transplant patients (*p* = 0.099). RVR was higher in kidney transplant patients than in the healthy controls and it reached a statistical significance (*p* = 0.01).

**Table 3:** Renal perfusion values in the healthy and in patients with kidney transplant

	Kidney transplant patients *N* = 19	Controls *N* = 10
Renal perfusion (ml min^−1^ g^−1^)	2.2 (2.0–3.0)	2.7 (2.4–4.0)
RVR (mmHg mL^− 1^min^− 1^g^− 1^)	47.0 (36.7–51.4)*	32.4 (24.6–39.6)

Values are median (Q1-Q3)

*RVR* Renal vascular resistance

*P < 0.05 controls versus kidney transplant patients

### Renal perfusion and other parameters in kidney transplant patients

Renal perfusion and RVR didn’t correlate with eGFR (*r* = 0.26, *p* = 0.28; *r* = − 0.13, *p* = 0.6, respectively). Both renal perfusion and RVR correlated with 24 h ambulatory systolic blood pressure (*r* = − 0.56, *p* = 0.016; *r* = 0.59, *p* = 0.008, respectively) and 24 h ambulatory pulse pressure (*r* = − 0.56, *p* = 0.016; *r* = 0.6, *p* = 0.008, respectively). There was a tendency to a correlation between renal perfusion and age (*r* = − 0.41, *p* = 0.08) and between RVR and transplant age (*r* = 0.41, *p* = 0.08). There was no correlation between renal perfusion or RVR and B-Hb, U-Na and fP-cholesterol (*p* > 0.05 in all).

### Renal perfusion and other parameters in the healthy

Renal perfusion and RVR correlated with eGFR in the healthy (*r* = 0.78, *p* = 0.0072; *r* = − 0.65, *p* = 0.041, respectively). There was a negative correlation between renal perfusion and age (*r* = − 0.68, *p* = 0.03). There was no correlation between renal perfusion or RVR and U-Na (*p* > 0.05).

### Doppler RI

Doppler RI of kidney transplants correlated statistically significantly with renal perfusion, RVR, age, pulse pressure on the PET-study day and 24 h ambulatory systolic blood pressure (*r* = − 0.51, *p* = 0.026; *r* = 0.59, *p* = 0.008; *r* = 0.46, *p* = 0.049; *r* = 0.66, *p* = 0.0023; *r* = 0.58, *p* = 0.012, respectively). There was no correlation between Doppler RI and eGFR or transplant age (*r* = − 0.005, *p* = 0.98; *r* = 0.1, *p* = 0.7).

### Histology of kidney transplants

Transplant biopsy was available in 17 of 19 patients.

The average number of glomeruli was 7 per biopsy. There were 2 interlobular arteries in 3 biopsies, 1 interlobular artery in 5 biopsies and 0 interlobular arteries in 11 biopsies. Sclerosed glomeruli were seen in 5 biopsies. The highest proportion of sclerosed glomeruli was 30%. None of the biopsies included arterial hyalinosis, arterial intimal thickening, intimal arteritis, peritubular capillaritis or double contour of glomerular basement membrane. No mesangial matrix expansion was seen. SV40T, CMV and C4d were all negative. More histological data is shown in Table 4.

**Table 4:** Histological findings of kidney transplant biopsies according to Banff classification

	Glomerulitis (g)	Interstitial inflammation(i)	Tubulitis (t)	Total inflammation (ti)	Interstitial fibrosis (ci)	Tubular atrophy (ct)
Banff score 0	13	9	15	8	12	12
1	1	6	1	7	5	5
2	1	1	1	1	0	0
3	2	1	0	1	0	0

Numbers in the table are kidneys

### Renal perfusion and fibrosis in kidney biopsy

There were 10 transplant biopsies in the group of no fibrosis (9 biopsies with score 0 and one biopsy with score 1) and 7 biopsies with mild fibrosis (5 biopsies with score 2, one biopsy with score 3 and one biopsy with score 4).

There was no statistically significant correlation between fibrosis and transplant perfusion (*p* = 0.56). Doppler RI was statistically significantly higher in the group of mild fibrosis than in the group of no fibrosis (*p* = 0.03). Twenty-four hour ambulatory MAP tended to be higher in the group of mild fibrosis than in the group of no fibrosis (*p* = 0.072). Comparison of renal perfusion, 24 h ambulatory MAP, transplant age and eGFR between the groups is presented in Table 5.

**Table 5:** Renal fibrosis and perfusion parameters

	eGFR (ml/min)	Renal perfusion (ml min^− 1^ g^− 1^)	Renal vascular resistance (mmHg mL^− 1^min^− 1^g^− 1^)	RI	24 h (mmHg)	Transplant age (months)
No fibrosis (*N* = 10)	55 (43–69)	2.3 (2.0–3.0)	40.9 (36.7–51.4)	0.66 (0.61–0.72)	98 (94–102)	44 (22–63)
Mild fibrosis (*N* = 7)	54 (47–71)	2.1 (1.8–2.4)	49.8 (44.2–58.4)	0.73 (0.70–0.76)*	106 (99–110)	27 (17–48)

Values are median (Q1-Q3)

*RI* Renal artery resistance index measured by Doppler ultrasound, *MAP* Mean arterial pressure

*P < 0.05, controls versus kidney transplant patients

### Renal perfusion and inflammatory changes in kidney biopsy

There were 9 biopsies in the group of no inflammatory changes (7 biopsies with score 0 and 2 biopsies with score 1), and 8 biopsies in the group of mild inflammatory changes (3 biopsies with score 2, 3 biopsies with score 5 and 2 biopsies with score 6). No statistically significant difference was found in eGFR, renal perfusion, RVR, 24 h MAP or Doppler RI between the groups (*p* > 0.05 in all). There was a statistically significant difference in age of transplant between the groups of mild inflammatory changes and of no inflammatory changes [57 (30–70) and 22 (17–48) months, respectively, *p* = 0.03]. RVR tended to be higher in the group of mild inflammatory changes than in the group of no changes [50 (46–61) and 39 (33–47) mmHg mL^− 1^ min^− 1^ g^− 1^, respectively, *p* = 0.05].

## Discussion

This is the first study assessing kidney transplant perfusion by non-invasive and quantitative PET-technique. Although cortical perfusion was equal between the healthy and the patients with kidney transplant (CKD stage 2–3), RVR of the patients was statistically significantly higher than that of the healthy. Furthermore, Doppler RI of transplants correlated with transplant perfusion and fibrosis. However, somewhat surprisingly, there was no correlation between transplant fibrosis and perfusion.

### Renal perfusion values in other studies

Renal cortical perfusion in the healthy was 2.7 (2.4–4.0) ml min^− 1^ g^− 1^ being similar with other studies by [^15^O]H_2_O PET, in which renal cortical perfusion in the healthy has varied between 1.6–4.7 ml min^− 1^ g^− 1^ [24, 25, 27, 29, 30, 40]. In our transplant patients the average eGFR was 57 (13) ml/min corresponding to moderate kidney impairment. In [^15^O]H_2_O PET based renal perfusion studies lower kidney perfusion has been demonstrated in patients with CKD than in the healthy [25, 27]. In our study, there was no statistically significant difference between renal cortical blood flow 2.2 (2.0–3.0) ml min^− 1^ g^− 1^ of transplanted kidneys and that of the healthy controls. However, CKD stage was more advanced in the patients of previous studies than in the patients of our study probably explaining the difference.

### Renal vascular resistance (RVR)

RVR describes the resistance to blood flow offered by renal blood vessels. Although renal perfusion values between the groups were the same RVR was higher in transplant patients than in healthy controls probably reflecting microvascular dysfunction in the kidneys of transplant patients. Because systolic blood pressure and MAP were statistically significantly higher in transplant patients than in controls kidney perfusion values didn’t differ between the groups. In other words, increased blood pressure maintained renal perfusion in transplant patients.

Hetzel et al. demonstrated similarly an increased RVR in transplant patients compared to controls by PAH (para-aminohippurate) – technique [41]. Also in their study renal perfusion was the same between the groups and blood pressure was statistically significantly higher in transplant patients than in controls.

There are several reasons for microvascular dysfunction in our study. Especially, calcineurin inhibitors are associated with increased vascular resistance [42, 43]. Persisting sympathetic overactivation after transplantation [44] may cause reduced perfusion due to vasoconstriction in kidney transplant. In our study transplant patients had clearly higher blood pressure than the healthy perhaps reflecting sympathetic overactivation. Finally, possible CKD - related microcirculatory changes like vascular rarefaction and endothelial dysfunction may explain increased vascular resistance [45].

### Transplant perfusion and histology

Our initial hypothesis was, that the decrease in perfusion would correlate to the changes in kidney transplant histopathology. However, we could not verify any correlation between transplant perfusion or RVR and fibrosis grade of kidney biopsy. Inflammatory changes in kidney biopsy and RVR tended to correlate.

In some Doppler based studies an inverse correlation between transplant perfusion and fibrosis has been shown [31, 32]. However, Schwenger [31] and Nankivell [32] used a non-quantitative sonographic technique. On the other hand, Pereira et al. showed an inverse correlation between transplant perfusion based on quantitative contrast-enhanced MRI and fibrotic changes [46]. The reason for different results might be, that in the study of Pereira et al. there was a higher grade of fibrosis in transplants compared to that of ours. The relatively small number of patients and the overall low biopsy fibrosis and inflammation scores were likely to contribute to the lack of correlation between transplant fibrosis and inflammation and perfusion in our study.

### Doppler RI of transplants

Transplant RI and perfusion correlated inversely in our study. Similar correlation has been shown early after transplantation [16]. Transplant RI also correlated with pulse pressure of 24 h ambulatory monitoring and recipient age. In accordance with these findings, Doppler RI is known to be influenced by several extrarenal factors like arterial blood pressure, vascular compliance, and age in transplant recipients [21, 47, 48]. Like transplant RI, also transplant perfusion correlated with pulse pressure of 24 h ambulatory monitoring, and recipient age. However, the latter didn’t reach a statistical significance.

Fibrosis of transplants seemed to increase in parallel with increasing Doppler RI. Other studies have demonstrated highly variable results concerning transplant histology and RI. Like in our study, Radermacher et al. showed a correlation between transplant fibrosis and transplant RI [20]. Gao et al. found a correlation between transplant fibrosis and Doppler flow velocities (end diastolic and peak systolic velocity) but not RI [19]. On the contrary to our findings, Naesens et al. showed no correlation between transplant fibrosis and RI in protocol biopsies [21].

### Kidney perfusion, RVR and eGFR

There was a statistically significant correlation between eGFR and perfusion and RVR in the healthy but not in the patients with kidney transplant. One reason for this discrepancy might be variable perfusion contribution from native kidneys in transplant patients although the effect of native kidneys on perfusion is known to decrease after transplantation [49].

### Limitations

There are some limitations in our study. Some transplant biopsies were not representative according to Banff criteria, especially arteries were partly lacking. However, the number of vessels should not have influenced the fibrotic and inflammatory scores that we were interested in. In addition, there was quite a long period between PET-imaging and kidney biopsy which may have influenced on our results. However, the average kidney function of patients was only mildly decreased during that time likely indicating that kidney biopsy histology would not have changed remarkably either. Furthermore, it is likely that cortical ROIs included an unknown admixture of medullary flow due to partial-volume effect and spatial resolution thus decreasing perfusion values. However, that phenomenon was similar both in the healthy and in the patients not influencing the difference in perfusion values between the groups. In addition, the state of autonomic nervous system which is known to influence renal perfusion [29] may have varied between the study subjects. However, it is not possible to exclude all factors affecting the autonomic nervous system. Finally, the number of study subjects was relatively small due to difficulties to find transplant patients without manifest CV disease.

## Conclusion

In conclusion, this pilot study showed the capability of PET-technique to measure kidney transplant perfusion. RVR of patients with kidney transplant was increased compared to healthy controls reflecting microvascular dysfunction of transplants. Further studies on larger number of transplant patients with advanced stage of CKD and varying degrees of fibrosis are needed to reveal the possible correlation between transplant fibrosis and perfusion.

## Supplementary information

**Additional file 1: Figure S1.** Aortic ROI of the healthy control subject.

**Additional file 2: Figure S2.** Iliac ROI of patient with kidney transplant.

**Additional file 3: Figure S3.** Image-derived arterial blood TACs from a control subject and a kidney transplant subject.

**Additional file 4: Figure S4.** Iliac artery ROIs in a three-dimensional PET image.

## Data Availability

The datasets used and/or analysed during the current study are available from the corresponding author on reasonable request.

## Abbreviations

ACE: Angiotensin converting enzyme
ARB: Angiotensin receptor blocker
CKD: Chronic kidney disease
CKD-EPI: The Chronic Kidney Disease Epidemiology Collaboration
CT: Computed tomography
HD: Hemodialysis
HR: Heart rate
MAP: Mean arterial pressure
MRI: Magnetic resonance imaging
PAH: Para-aminohippurate
PD: Peritoneal dialysis
PET: Positron emission tomography
p_phys_: partition coefficient
RBF: Renal blood flow
RI: Resistance index
ROI: Regions of interest
RVR: Renal vascular resistance
TAC: Time activity curve
