# Supplementary material for: Renal vascular resistance is increased in patients with kidney transplant
Source: BMC Nephrol. 2019 Nov 27;20:437. doi: 10.1186/s12882-019-1617-2 (PMC6882025; doi:10.1186/s12882-019-1617-2)
Supplement: Supplementary file 1 — Additional file 1: Figure S1. Aortic ROI of the healthy control subject. [file 12882_2019_1617_MOESM1_ESM.docx]

Additional file 1: **Figure S1.** Aortic ROI of the healthy control subject.


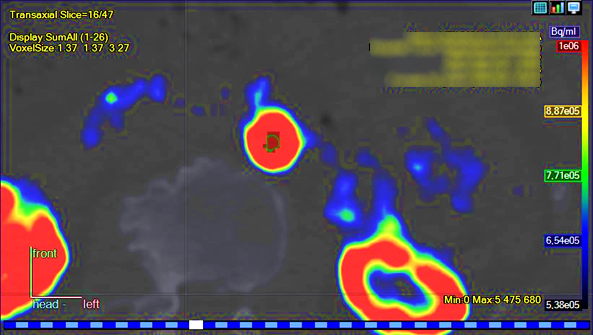


Gray arrow points to aortic ROI**.**
