# Supplementary material for: Renal vascular resistance is increased in patients with kidney transplant
Source: BMC Nephrol. 2019 Nov 27;20:437. doi: 10.1186/s12882-019-1617-2 (PMC6882025; doi:10.1186/s12882-019-1617-2)
Supplement: Supplementary file 2 — Additional file 2: Figure S2. Iliac ROI of patient with kidney transplant. [file 12882_2019_1617_MOESM2_ESM.docx]

Additional file 2: **Figure S2.** Iliac ROI of patient with kidney transplant


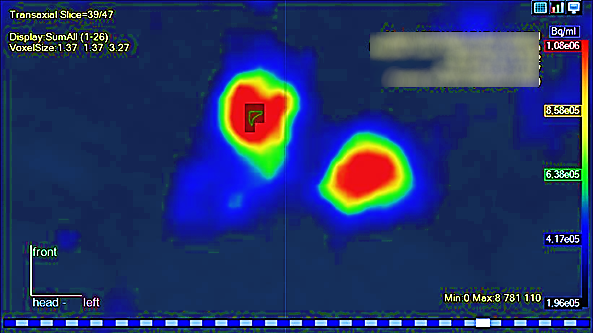


Black arrow points to ROI-area in right external iliac artery.
