# Supplementary material for: Renal vascular resistance is increased in patients with kidney transplant
Source: BMC Nephrol. 2019 Nov 27;20:437. doi: 10.1186/s12882-019-1617-2 (PMC6882025; doi:10.1186/s12882-019-1617-2)
Supplement: Supplementary file 3 — Additional file 3: Figure S3. Image-derived arterial blood TACs from a control subject and a kidney transplant subject. [file 12882_2019_1617_MOESM3_ESM.docx]

Additional file 3: **Figure S3.** Image-derived arterial blood TACs from a control subject and a kidney transplant subject.

For control subjects the ROI was drawn on abdominal aorta, but in kidney transplant patients only iliac artery was visible in the images. Concentrations are corrected for decay of O-15 and given in standardized uptake value (SUV) units (scaled for injected dose of radioactivity and the mass of subject). The image-derived input function was not corrected for dispersion, because of its location was close to the kidney. Recovery correction for the image-derived input curves was not considered necessary, because very thin ROIs were placed in the iliac arteries, and image reconstruction parameters had been modified to produce similar blood curves from abdominal aorta and iliac arteries than in left ventricular cavity of the heart.
