# Supplementary material for: Renal vascular resistance is increased in patients with kidney transplant
Source: BMC Nephrol. 2019 Nov 27;20:437. doi: 10.1186/s12882-019-1617-2 (PMC6882025; doi:10.1186/s12882-019-1617-2)
Supplement: Supplementary file 4 — Additional file 4: Figure S4. Iliac artery ROIs in a three-dimensional PET image. [file 12882_2019_1617_MOESM4_ESM.docx]

Additional file 4: **Figure s4.** Iliac artery ROIs in a three-dimensional PET image


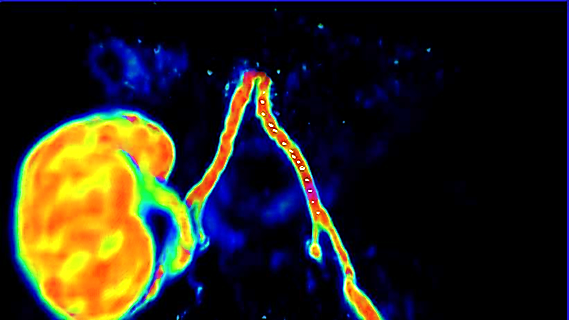


Blue arrow points to ROI:s in left external iliac artery.
